# Efficacy and Safety of Flexible and Navigable Suction Ureteral Access Sheath Versus Conventional Ureteral Access Sheath in Retrograde Intrarenal Surgery: An Updated Systematic Review and Meta-Analysis

**DOI:** 10.3390/medicina62030536

**Published:** 2026-03-13

**Authors:** Seok Cho, Joo Yong Lee, Hae Do Jung, Min Gu Park

**Affiliations:** 1 Department of Urology, Inje University Ilsan Paik Hospital, Inje University College of Medicine, Goyang 10380, Republic of Korea; seokcho@paik.ac.kr; 2 Department of Urology, Severance Hospital, Urological Science Institute, Yonsei University College of Medicine, Seoul 03722, Republic of Korea; joouro@yuhs.ac; 3 Department of Urology, Korea University Anam Hospital, Korea University College of Medicine, Seoul 02841, Republic of Korea

**Keywords:** flexible ureteroscopy, suction ureteral access sheath, kidney stones, meta-analysis

## Abstract

*Background and Objectives*: Ureteral access sheaths (UASs) are widely used in retrograde intrarenal surgery (RIRS) to facilitate irrigation and instrument access. Recently, flexible and navigable suction UASs (FANS-UASs) have been developed to enhance visibility and stone fragment evacuation; however, their comparative effectiveness remains uncertain. This study aimed to evaluate the clinical outcomes of FANS-UAS versus conventional UAS during RIRS for renal stones. *Materials and Methods*: A systematic review and meta-analysis were performed following PRISMA guidelines. PubMed, Embase, and the Cochrane Library were searched through May 2025 for comparative studies of FANS-UAS and conventional UAS. Study quality was assessed using the Scottish Intercollegiate Guidelines Network checklist. Primary outcomes included stone-free rate (SFR), operative time, complications, and hospital stay. Subgroup analyses were conducted according to stone size (≤2 cm vs. >2 cm). *Results*: Nine studies involving 1791 patients were included. FANS-UAS demonstrated a significantly higher SFR (OR = 5.99, 95% CI: 2.86–12.51; I^2^ = 86.7%) and fewer complications (OR = 0.33, 95% CI: 0.23–0.45; I^2^ = 0%). Operative time and hospital stay did not differ significantly between groups. Subgroup analysis showed no significant SFR difference for stones ≤2 cm, whereas for stones >2 cm, FANS-UAS tended to yield higher SFR—though based on limited evidence. *Conclusions*: FANS-UASs appear to improve stone clearance and reduce perioperative complications in RIRS without increasing operative burden. While further high-quality randomized trials are needed, current evidence supports the growing adoption of FANS-UAS in endourological practice.

## 1. Introduction

Urolithiasis is a prevalent urological condition with increasing global incidence, necessitating the development of efficient and minimally invasive therapeutic approaches. The prevalence of urinary stones varies widely, ranging from 1% to 20% [1], and in countries with a high standard of life, renal stone prevalence is notably high (>10%). In some regions, an increase of >37% over the last 20 years has been reported [2,3,4]. Retrograde intrarenal surgery (RIRS) has become a key method for treating small-to-medium-sized kidney stones because it is partially invasive and yields favorable clinical outcomes [5,6]. Using ureteral access sheaths (UASs) during RIRS has become a common practice because it allows for repeated access to the collecting system, lowers intrarenal pressure, improves irrigation, and shortens operative time by making it easier to obtain stone fragments [5,6].

Despite these advantages, conventional UASs exhibit several limitations. They often have difficulty maintaining the right amount of pressure in the kidneys during long procedures, which can cause pyelovenous backflow. This increases the risk of infectious complications. Additionally, the passive properties of conventional sheaths do not provide sufficient support for effective fragment removal, which lowers the stone-free rate (SFR) and lengthens surgery time [7].

**Figure 1:**
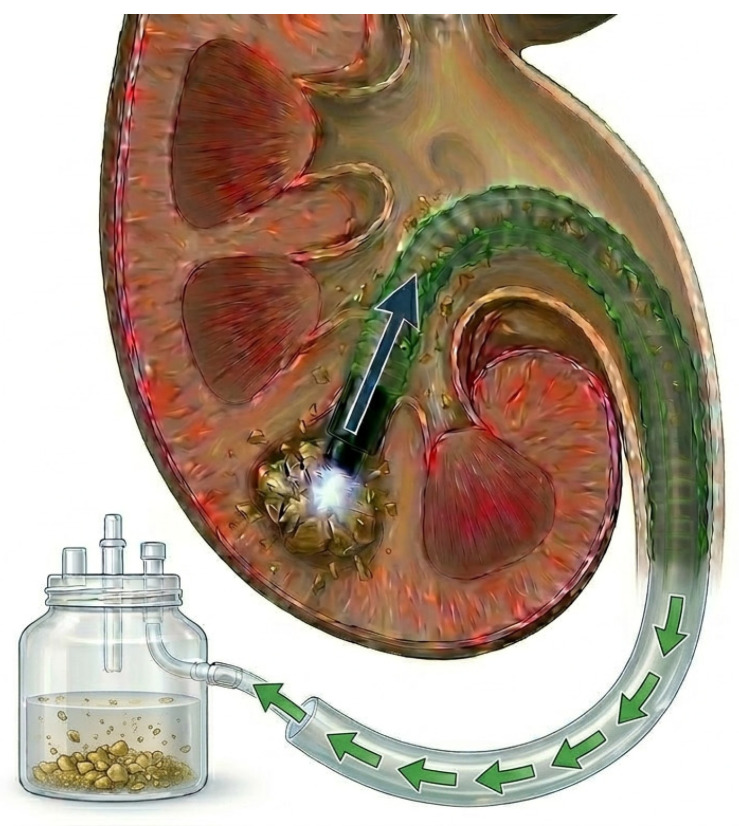
Schematic illustration of flexible and navigable suction ureteral access sheath.

Flexible and navigable suction ureteral access sheath (FANS-UAS) was recently designed to overcome these problems (Figure 1). FANS-UAS combines active suction mechanisms with better mobility to facilitate intraoperative visualization, maintain low intrarenal pressure, and facilitate the active removal of stone pieces [8,9]. Although several clinical studies [10,11,12,13,14,15,16,17,18] and systematic reviews [19,20,21,22,23] have compared the safety and efficacy of FANS-UAS and conventional UAS, their findings remain heterogeneous. Therefore, we performed an updated meta-analysis incorporating a subgroup analysis according to stone size to elucidate size-dependent outcomes and improve the precision of pooled estimates. This study aimed to comprehensively compare SFR, operative duration, complication rates, and length of hospital stay, thereby providing a more robust evidence base to guide therapeutic decision-making in RIRS.

## 2. Materials and Methods

### 2.1. Inclusion Criteria and Exclusion Criteria

Inclusion criteria for this study were as follows: (a) patients with renal stones or upper ureter stones; (b) comparison of FANS-UAS and conventional UAS in patients undergoing RIRS; (c) outcome measures, including SFR, complications, operative time, and hospital stay. Published studies were excluded if the full text was not available. The statement on Preferred Reporting Items for Systematic Reviews and Meta-Analyses was followed in preparing this report (Supplementary Table S1) [24]. This systematic review was not subject to evaluation by the ethics committee or institutional review board, as systematic reviews and meta-analyses do not necessitate prior approval.

### 2.2. Search Strategy

A systematic review was conducted to identify pertinent articles comparing treatments for renal stones utilizing the three English language databases, PubMed, EMBASE, and the Cochrane Library, up to May 2025. Search strategies were established to include medical subject headings keywords such as “flexible ureteral access sheath”, “suction sheath”, “FANS”, “retrograde intrarenal surgery”, “RIRS”, and combinations of these search terms.

### 2.3. Study Selection and Data Extraction

Two researchers (S.C. and H.D.J.) independently screened the titles and abstracts of identified articles using the search strategy to eliminate irrelevant studies. They also evaluated the complete text of the articles for pertinence. Relevant articles were extracted from each study, encompassing details such as author, year of publication, country, study design, patient characteristics, treatments, and outcome variables including “SFR”, “complication rate”, “operative time”, and “hospital stay”.

### 2.4. Study Quality Assessment

We employed the Cochrane Risk of Bias (ROB) tool for randomized controlled trials (RCT) and the Methodological Index for Non-Randomized Studies (MINORS). The quality of evidence was assessed independently by our researchers (S.C. and H.D.J.) utilizing the Scottish Intercollegiate Guidelines Network (SIGN) checklist, which encompasses multiple study types, including systematic reviews and meta-analyses, RCTs, cohort studies, case–control studies, diagnostic studies, and economic studies. All disputes concerning quality assessment outcomes were settled following a discussion with a third reviewer (M.G.P.).

### 2.5. Statistical Analysis

Odds ratios (ORs) and 95% confidence intervals (CIs) were computed and presented as dichotomous variables. The weighted mean difference (MD) and 95% confidence interval (CI) were computed for the continuous variables. The Chi-squared test with a *p*-value of 0.05 was employed to assess statistical heterogeneity, while the I^2^ statistic was utilized to measure the extent of heterogeneity [25]. If the reported I^2^ statistic was less than 50%, we employed a common (fixed) effects model; otherwise, a random-effects model was utilized. The Higgins I^2^ statistic was computed as follows:

$$I^{2}=\frac{Q-df}{Q}\times 100\%$$

where “Q” denotes the Cochrane heterogeneity statistic and “df” represents the degrees of freedom. All meta-analyses were conducted using the meta and metasens packages in R software, version 4.1.3 (R Foundation for Statistical Computing, Vienna, Austria; http://www.r-project.org), in addition to Cochrane’s Review Manager (RevMan Web). This systematic review was registered in PROSPERO (CRD420251069865).

## 3. Results

### 3.1. Eligible Studies

**Figure 2:**
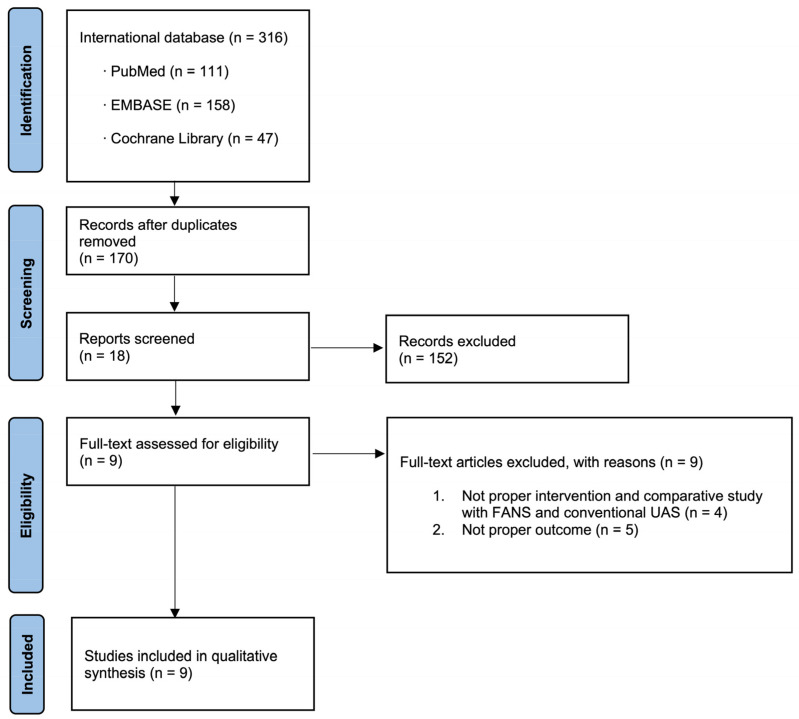
Study flow chart.

In total, 316 studies were included. Nine articles were identified that were related to the current study and selected for inclusion in the meta-analysis following a comprehensive review of the literature (Figure 2).

### 3.2. Characteristics of the Included Studies

Nine studies were included in the meta-analysis, comprising two randomized controlled trials (RCTs) [10,18] and seven non-randomized (one prospective and six retrospective) [11,12,13,14,15,16,17]. These comparative studies described patients who underwent RIRS using FANS-UAS and conventional UAS for renal or proximal ureteral stones. The included studies were published between September 2023 and March 2025. Among these studies, two were multicenter studies, one was conducted across China, Turkey, Malaysia, and the Philippines [18] and another across Singapore, the United Kingdom, Canada, Saudi Arabia, India, Indonesia, Russia, Hong Kong, Italy, and France [14]. Of the single-center studies, five were conducted in China [11,12,13,16,17], one in Italy [10], and one in Turkey [15]. Characteristics of the nine included studies are presented in Table 1 [10,11,12,13,14,15,16,17,18].

### 3.3. Quality Assessment

The methodological quality of the included studies was assessed utilizing the SIGN checklist. Among the two RCTs, one study demonstrated a low ROB and was graded as SIGN 1+, as it clearly described randomization, allocation concealment, and objective outcome assessment using computed tomography (CT) imaging [18]. In contrast, Cacciatore et al. [10] was graded as SIGN 1− due to unclear allocation concealment and lack of blinding, indicating a higher ROB despite being randomized. Of the seven non-randomized studies, four studies were evaluated as SIGN 2+, indicating well-conducted cohort studies with a relatively low ROB [13,14,15,16]. These studies used appropriate control groups, reported comparable baseline characteristics, and used either propensity score matching or multicenter designs to minimize selection bias. The remaining three observational studies were classified as SIGN 2−, mainly due to retrospective single-center designs, the absence of matching or adjustment for confounders, and potential selection bias [11,12,17].

### 3.4. Risk of Bias Assessment

**Figure 3:**
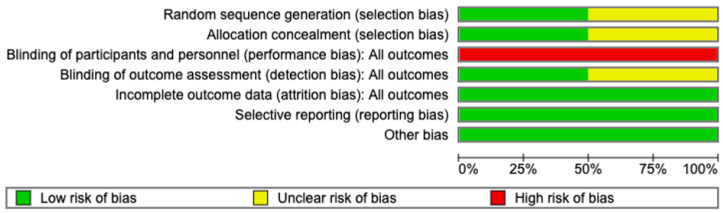
Risk of bias graph.

**Figure 4:**
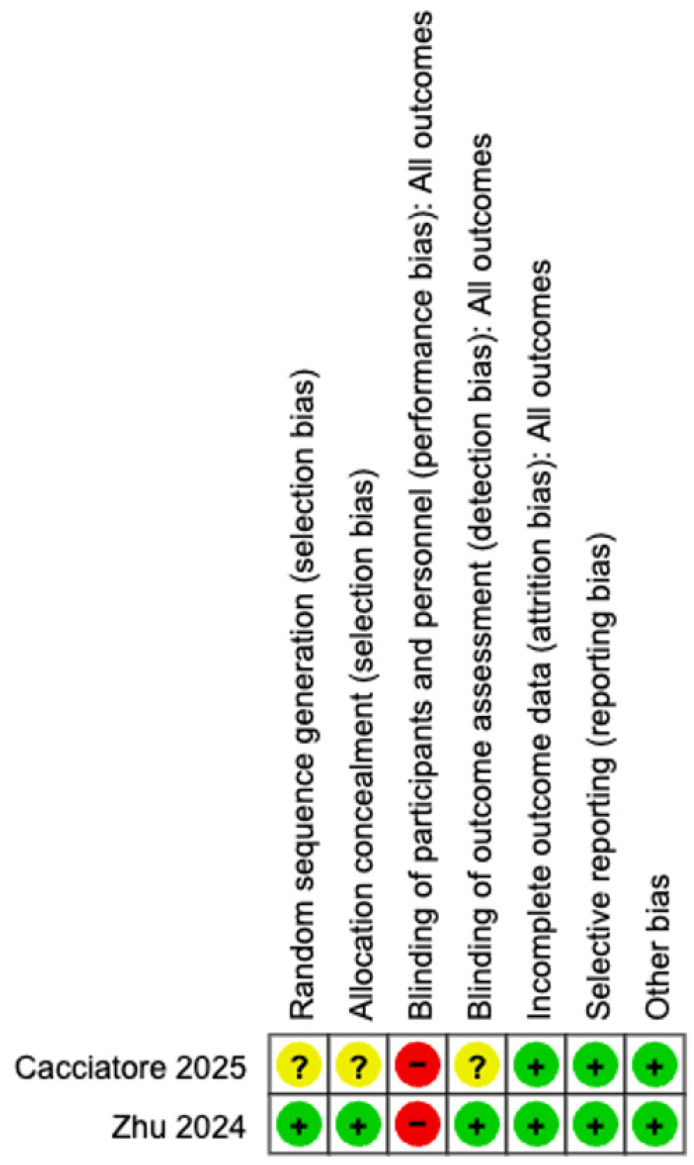
Risk of bias summary [10,18].

The ROB for the RCTs is shown in Figure 3 and Figure 4. ROB in the included RCTs was evaluated using the Cochrane RoB tool. Two RCTs were included in this assessment [10,18]. Overall, Zhu et al. [18] demonstrated a low ROB across all major domains, whereas Cacciatore et al. [10] showed uncertainty or high risk in several key areas, resulting in comparatively lower methodological quality. Zhu et al. [18] assessed random sequence generation (selection bias) as low risk because of the explicit description of computerized randomization, whereas Cacciatore et al. [10] did not provide sufficient details, leading to an unclear risk rating. Allocation concealment was adequately performed by Zhu et al. [18], but remained unclear in the study by Cacciatore et al. [10]. Both studies exhibited a high risk of performance bias (blinding of participants and personnel), which was expected given the nature of the surgical interventions and the visibility of different UAS devices. However, blinding of the outcome assessment (detection bias) was adequately addressed by Zhu et al. [18] through a blinded radiologic imaging review, whereas Cacciatore et al. [10] did not clearly report the assessor blinding and was rated as unclear. In terms of attrition bias (incomplete outcome data) and reporting bias (selective reporting), both RCTs were rated as low risk, with complete follow-up and consistent reporting of all predefined outcomes. No other sources of bias were identified. The MINORS scores for the non-RCTs are shown in Table 2. Seven non-randomized studies reported total MINORS scores between 17 and 22. This indicates that the overall methodological quality of the included studies was moderate to good. However, nearly all studies lacked prospective data collection. Despite some limitations, the statistical analyses were generally adequate across the studies.

### 3.5. Publication Bias

**Figure 5:**
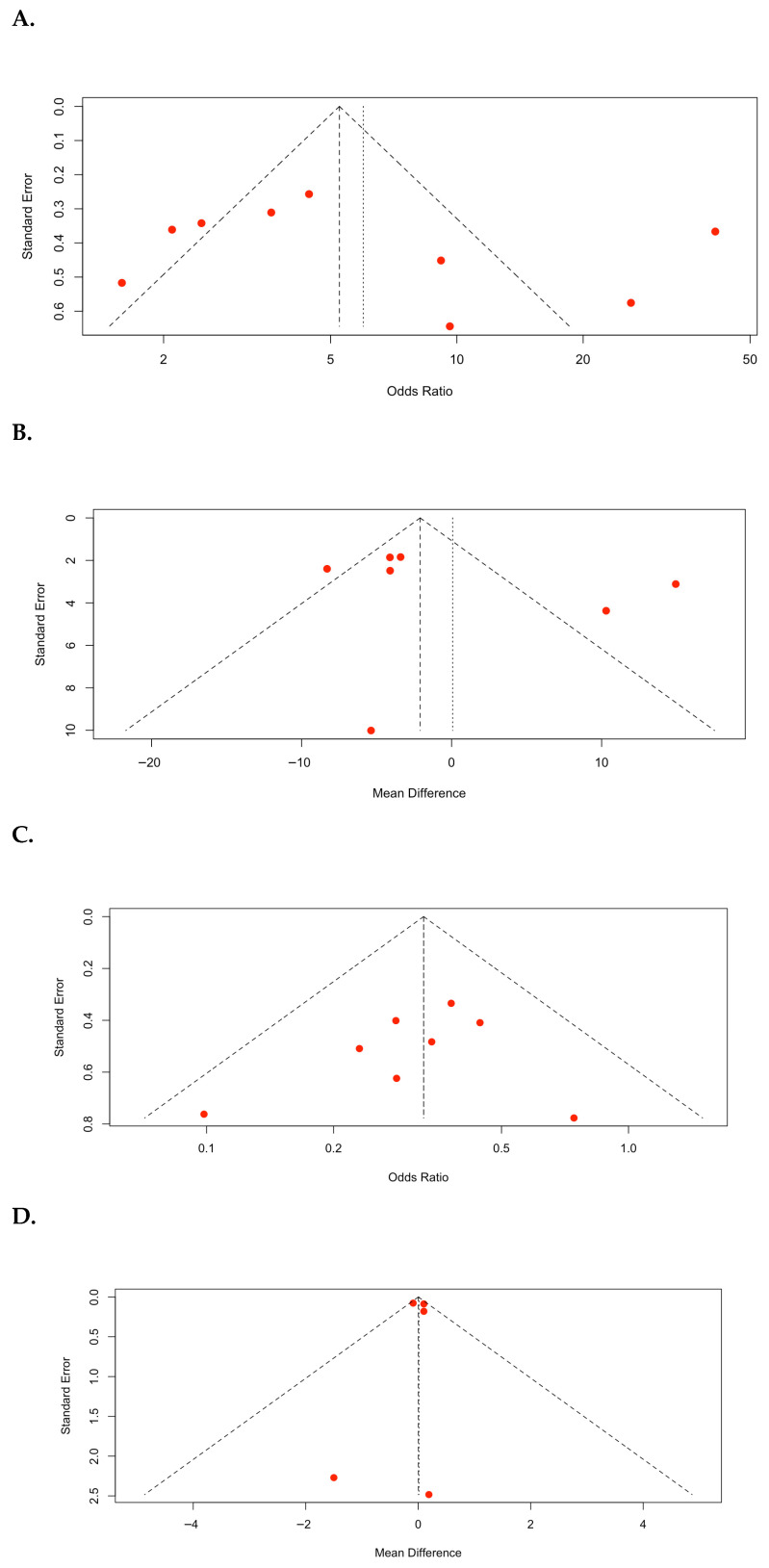
Funnel plots: (**A**) stone-free rate (SFR); (**B**) operative time; (**C**) complication rate; (**D**) hospital stay.

Funnel plots of the meta-analyses are shown in Figure 5. In the SFR (Figure 5A), a visual inspection of the plot revealed a relatively symmetrical distribution of studies around the pooled effect size. However, a small degree of asymmetry was observed, indicating the small effect of the study. However, the overall pattern did not strongly suggest a significant publication bias. Statistical tests for asymmetry (Egger’s regression test: *p* = 0.4916) were not significant, indicating no evidence of small-study bias. For operative time (Figure 5B), the distribution of studies appeared relatively symmetrical around the pooled MD; although two studies showed a higher MD with a larger standard error, the overall pattern did not demonstrate clear evidence of asymmetry. Statistical tests for asymmetry (Egger’s regression test: *p* = 0.4916) were not significant, indicating no evidence of small-study bias. For complication rate (Figure 5C), the plot demonstrated a visually symmetrical distribution of studies around the pooled odds ratio. Statistical tests for asymmetry (Egger’s regression test, *p* = 0.4608) were not significant, implying that publication bias was unlikely to have had a meaningful effect on the pooled estimate of complication rates. During hospital stay (Figure 5D), the plot demonstrated a highly symmetrical distribution of studies around the pooled MD. Most studies clustered near the top of the funnel, indicating relatively small standard errors and high precision. Statistical tests for asymmetry (Egger’s regression test: *p* = 0.9379) were not significant, indicating no strong evidence of small-study bias.

### 3.6. Stone-Free Rate

**Figure 6:**
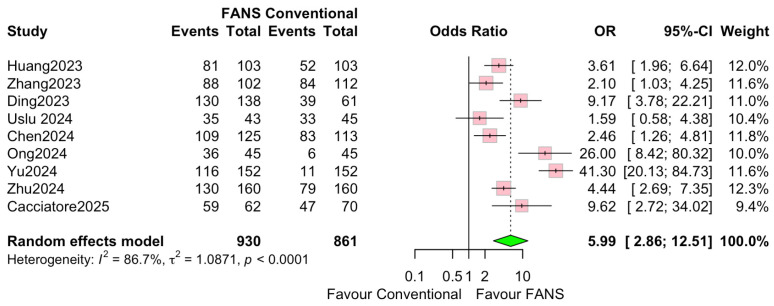
Forest plots: Comparison of FANS-UAS and conventional UAS for SFR [10,11,12,13,14,15,16,17,18].

Nine studies involving 1791 patients (930 and 861 in the FANS-UAS and conventional UAS groups, respectively) were included in the meta-analysis evaluating SFR [10,11,12,13,14,15,16,17,18]. As shown in Figure 6, the pooled results using a random-effects model demonstrated that FANS-UAS significantly increased the likelihood of achieving SFR compared with conventional UAS (OR = 5.99, 95% CI = 2.86–12.51, *p* < 0.0001). Across individual studies, all included datasets favored FANS-UAS. The odds ratio was between 2.10 and 41.30, indicating a consistent benefit across varying surgical settings and populations. No study demonstrated superiority of conventional UAS over FANS-UAS. Substantial inter-study heterogeneity was observed (I^2^ = 86.7%, τ^2^ = 1.026), reflecting differences in stone size, surgical technique, imaging modalities used for SFR assessment, surgeon experience, and variations in FANS-UAS device design. Despite this, the overall effect consistently favored FANS-UAS, supporting the robustness of the pooled outcome.

### 3.7. Operative Time

**Figure 7:**
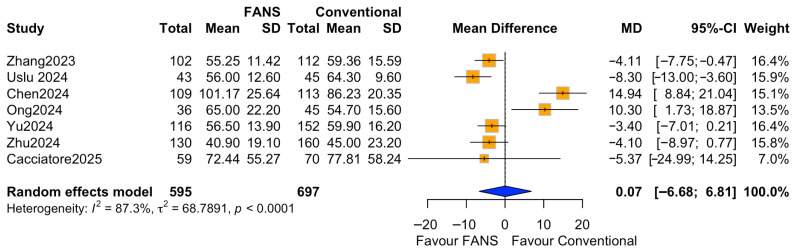
Forest plots: comparison of operative time between FANS-UAS and conventional UAS [10,11,14,15,16,17,18].

Seven studies comprising 1292 patients (595 and 697 in the FANS-UAS and conventional UAS groups, respectively) reported operative time [10,11,14,15,16,17,18]. Pooled analysis using a random-effects model showed no statistically significant difference in mean operative time between FANS-UAS and conventional UAS (MD = 0.07, 95% CI = −6.68 to 6.81, *p* = 0.984), as shown in Figure 7. At the individual study level, four studies demonstrated numerically shorter operative times in the FANS-UAS group [10,16,17,18], although only Zhang et al. [17] (MD = −4.11, 95% CI = −7.75 to −0.47) reached statistical significance. In contrast, Chen et al. and Ong et al. [11,14] reported significantly longer operative times in the FANS-UAS group (MD = 14.94, 95% CI = 8.84–21.04; MD = 10.30, 95% CI = 1.73–18.87). There was substantial heterogeneity across the included studies (I^2^ = 87.3%, τ^2^ = 66.68, *p* < 0.0001), likely attributable to differences in stone burden, laser settings, suction system design, surgeon experience, and surgical strategies.

### 3.8. Complication Rate

**Figure 8:**
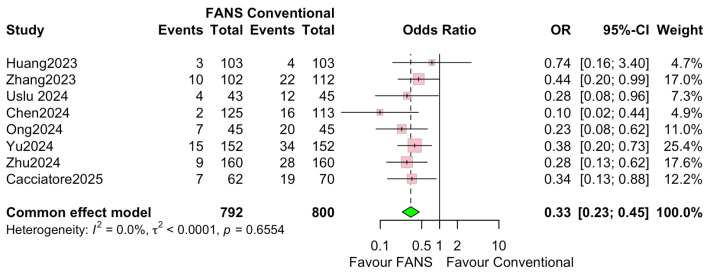
Forest plots: comparison of complication rate between FANS-UAS and conventional UAS [10,11,13,14,15,16,17,18].

Eight studies, involving 1592 patients (792 and 800 in the FANS-UAS and conventional UAS groups, respectively), reported postoperative complication rates [10,11,13,14,15,16,17,18]. As shown in Figure 8, the pooled analysis using a common effects model demonstrated that FANS-UAS was associated with a significantly lower risk of complications compared with conventional UAS (OR = 0.33, 95% CI = 0.23–0.45, *p* < 0.0001). Across individual studies, seven of eight trials favored FANS-UAS, with ORs consistently below 1.0. No study reported higher complication rates in the FANS-UAS group. The most reported complications were Clavien–Dindo grades I–II, which included fever, hematuria, transient pain, or infection. No significant heterogeneity was detected (I^2^ = 0.0%, *p* = 0.6554), indicating strong consistency among the included studies, despite differences in study design, surgeon experience, and FANS-UAS device type.

### 3.9. Hospital Stay

**Figure 9:**
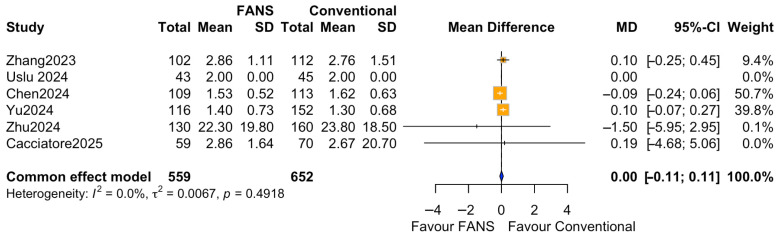
Forest plots: comparison of hospital stay between FANS-UAS and conventional UAS [10,11,15,16,17,18].

Six studies (1211 patients), comprising 559 patients in the FANS-UAS group and 652 patients in the conventional UAS group, reported the duration of postoperative hospital stay [10,11,15,16,17,18]. As shown in Figure 9, the pooled analysis using a common effect model revealed no significant difference in hospital stay between FANS-UAS and conventional UAS (MD = 0.00, 95% CI = −0.11 to 0.11, *p* = 0.9604). None of the included studies demonstrated a statistically significant difference in length of stay at the individual level. Heterogeneity was negligible (I^2^ = 0.0%, *p* = 0.5234), indicating high consistency among study results.

### 3.10. Subgroup Analysis According to Stone Size

**Figure 10:**
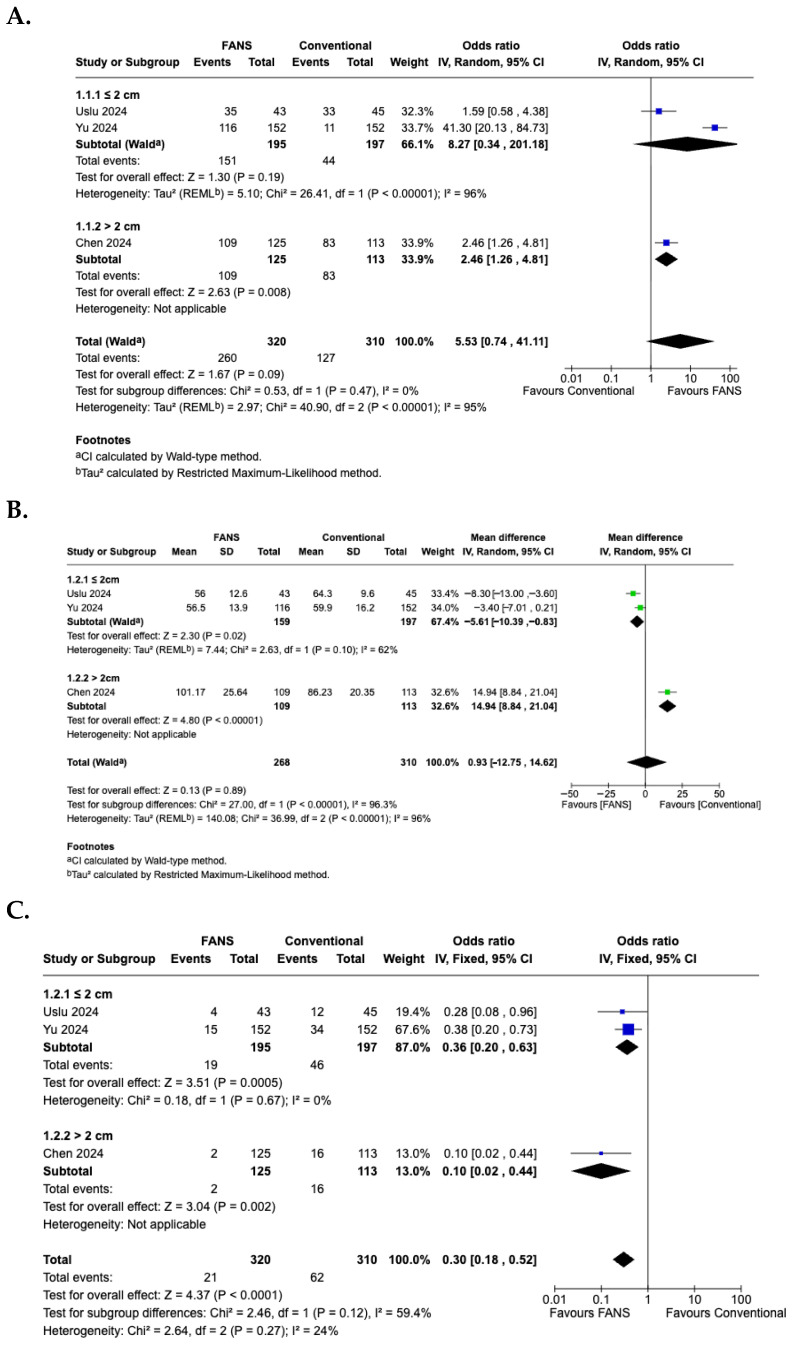

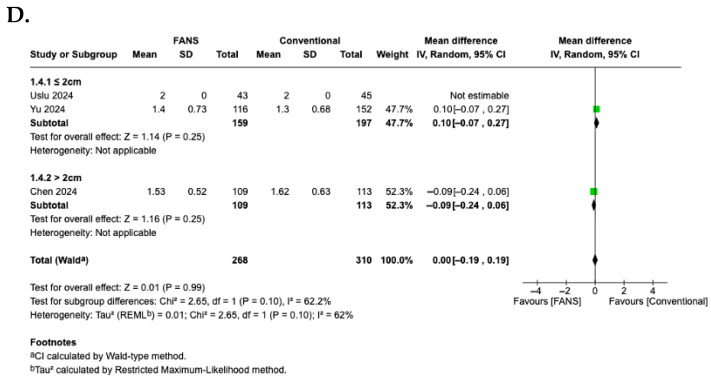
Forest plots—subgroup analysis according to stone size: (**A**) SFR; (**B**) operative time; (**C**) complication rate; (**D**) hospital stay [11,15,16].

A subgroup analysis based on stone size was performed to evaluate differences in efficacy and safety between FANS-UAS and conventional UAS. For the SFR (Figure 10A), the subgroup of studies with stones ≤2 cm demonstrated no significant difference between FANS-UAS and conventional UAS (OR = 8.27, 95% CI = 0.34–201.18, *p* = 0.19), with substantial heterogeneity (I^2^ = 96%) [15,16]. In contrast, the study involving stones >2 cm showed a significantly higher stone-free rate in the FANS-UAS group (OR = 2.46, 95% CI = 1.26–4.81, *p* = 0.008) [11]. These findings suggest that FANS-UAS may provide greater benefit in cases with larger stone burdens. For operative time (Figure 10B), the subgroup of studies with stones ≤2 cm showed a significantly shorter operative time in the FANS-UAS group (MD = −5.61, 95% CI = −10.39 to −0.83, *p* = 0.02). In contrast, the study involving stones >2 cm showed a significantly longer operative time in the FANS-UAS group (MD = 14.94, 95% CI = 8.84 to 21.04, *p* < 0.001) [11]. For complication rate (Figure 10C), FANS-UAS was associated with significantly fewer complications compared with conventional UAS for stones ≤2 cm (OR = 0.36, 95% CI = 0.20–0.63, *p* = 0.0005; I^2^ = 0%) [15,16]. Similarly, in the >2 cm subgroup, FANS-UAS demonstrated a substantial reduction in complications (OR = 0.10, 95% CI = 0.02–0.44, *p* = 0.002) [11]. For hospital stay (Figure 10D), there was no significant difference between FANS-UAS and conventional UAS in both subgroups, including stones ≤2 cm as well as those >2 cm. However, this interpretation should be approached with caution, as the subgroup representing stones >2 cm was based on a single study [11], limiting the generalizability of this result.

## 4. Discussion

The present meta-analysis provides a comprehensive synthesis of the comparative efficacy and safety of FANS-UAS and conventional UAS during RIRS. Compared with conventional UAS across studies, FANS-UAS demonstrated a significantly higher SFR and reduced postoperative complications while maintaining a comparable operative time and hospital stay to conventional UAS. This benefit may be attributed to improved intrarenal pressure control and continuous suction of debris and irrigation fluid. In terms of operative time, high heterogeneity indicates that operative efficiency may be influenced by patient selection and operator-dependent factors rather than the sheath type alone. Comparable hospital stay results between FANS-UAS and conventional UAS confirm that the addition of suction functionality does not increase postoperative recovery burden. This finding suggests that despite improvements in SFR and complication reduction, postoperative recovery duration and discharge timing are likely to be more influenced by institutional protocols, pain control, infection prophylaxis, and local healthcare policies than by the type of UAS used. Subgroup analyses indicated that the relative benefits of FANS-UAS may differ according to stone size. For stones ≤2 cm, FANS-UAS did not improve SFR compared with conventional UAS, although it was associated with a significantly lower complication rate. In contrast, for stones >2 cm, the available study suggested that FANS-UAS achieved a higher SFR and markedly fewer complications, implying that its advantages may become more pronounced in the setting of larger stone burdens. Operative time also showed a size-dependent pattern, with shorter procedures in the FANS-UAS group for ≤2 cm stones but longer operative times for >2 cm stones. Length of hospital stay did not differ between the groups in either subgroup. In larger or complex stones (>2 cm), FANS-UAS enables more efficient dust evacuation, potentially reducing the need for secondary procedures such as repeat RIRS or percutaneous nephrolithotomy [26,27]. The improvement in SFR for stones >2 cm should be interpreted cautiously, as this finding was derived from a single retrospective study and therefore requires confirmation in future randomized or multicenter comparative trials.

Compared with previous meta-analyses [19,20], the current study provides an improvement by incorporating the study by Cacciatore et al. [10], a well-conducted multicenter RCT from Italy, thereby strengthening the evidence base and global applicability of the findings beyond the previously predominant Asian data pool. Additionally, compared with a previous meta-analysis [21] that synthesized 34 studies encompassing both comparative and non-comparative designs with considerable methodological heterogeneity, our meta-analysis achieved greater methodological rigor by restricting inclusion to comparative studies only. This selective approach enhanced internal validity and minimized potential population overlap, which may have influenced previous analyses that incorporated descriptive series from overlapping international consortia. Additionally, a key distinction of our meta-analysis was the incorporation of a subgroup analysis based on a 2 cm threshold for stone size. Although Wang et al. [22] similarly stratified outcomes according to stone size, their dataset included several studies in which the maximum stone diameter was not clearly reported, but was nonetheless assigned to specific size-based subgroups. Additionally, the inclusion of the study by Vaddi et al. [28], a non-peer-reviewed preprint, further increases uncertainty regarding the reliability of their subgroup conclusions.

The superiority of FANS-UAS can be elucidated in terms of hydrodynamic efficiency, visual field optimization, and infection prevention. Unlike conventional UAS, which maintain a passive open channel for irrigation without the ability to actively regulate intrarenal pressure, FANS-UAS incorporates an integrated negative-pressure channel that allows for continuous evacuation of fluid and dust during laser lithotripsy, thereby maintaining intrarenal pressure at safe physiological levels (often <40 mmH_2_O) [19,29,30,31]. This constant suction effect, sometimes described as the “vacuum cleaner effect”, efficiently removes fine dust and stone fragments in real time, thereby preventing intermittent spikes in intrapelvic pressure that could otherwise exceed 300 mmH_2_O and lead to pyelovenous backflow, which increases the risk of postoperative fever or sepsis due to systemic dissemination of bacteria and endotoxins. The bendable distal segment of FANS-UAS enables the sheath to conform to the angulation of the flexible ureteroscope, thereby increasing access to calyceal recesses that rigid sheaths cannot reach; this facilitates a more complete evacuation of fragments, higher SFR, and reduced need for auxiliary procedures [19,29,31]. Hydrodynamic and clinical studies demonstrated that FANS-UAS sustained continuous flow without compromising visibility, even under high irrigation rates, while real-time aspiration reduces laser defocusing attributable to floating fragments, enhancing ablation precision and reducing operative time. Clinically, these advantages result in fewer episodes of pyelovenous backflow, lower infection rates, thermal injury, and reduced hematuria severity, as repeatedly demonstrated in Asian and Western trials [30,31,32,33].

For endourologists, FANS-UAS translates into a more controlled surgical environment, better visualization, and less dependence on accessory tools such as baskets [30,34]. Additionally, because FANS-UAS is compatible with standard flexible ureteroscopes and suction systems, its integration into surgical workflows is straightforward and requires minimal training or equipment modification [31,34]. Moreover, improved visibility during laser lithotripsy enhances procedural safety, allowing finer laser modulation and minimizing inadvertent mucosal injury [11,18,30,35]. Collective evidence supports routine adoption of FANS-UAS as the preferred sheath for RIRS, particularly in cases involving high stone burden, infection-prone patients, or prolonged lithotripsy [10,34,36].

Despite these robust findings, several limitations of this study must be acknowledged. First, although the inclusion of two multicenter RCTs considerably improves the evidence quality, most of the included studies remained retrospective, which introduces potential selection and reporting biases. Second, although Egger’s regression tests did not detect significant publication bias, the relatively small number of included studies may limit the statistical power of this method. Therefore, the absence of statistical evidence for publication bias should be interpreted with caution. Third, device heterogeneity exists: different FANS-UAS models (NTFS-UAS, S-UAS, FANS, and ClearPetra^®^) use variable suction pressures and tip flexibility degrees, which may subtly affect outcomes. Fourth, not all the studies standardized the definition of “SFR”, with some using CT at 1 month and others, including KUB or ultrasound, at 1 day, thereby introducing heterogeneity in the endpoint measurement. Fourth, very few studies directly measured intraoperative intrarenal pressure, making it difficult to objectively quantify physiological differences. Fifth, subgroup analysis showed no significant SFR difference for stones ≤2 cm, whereas for stones >2 cm, FANS-UAS tended to yield a higher SFR, though this observation was based on limited evidence. This potential advantage requires further confirmation in larger comparative studies. Finally, most trials included short-term follow-up (≤3 months), leaving the long-term durability of stone-free status or potential ureteral stricture risks unknown. Nonetheless, the consistency of outcomes across diverse study designs and geographic regions suggests that the overall findings are relatively robust; however, these results should be interpreted cautiously given the limited long-term follow-up and the lack of direct physiological measurements such as intrarenal pressure. Future studies should focus on the following key topics. RCTs should incorporate continuous intrarenal pressure monitoring to quantitatively validate hypothesized pressure-lowering effect of FANS-UAS, while engineering-based comparative analyses focusing on suction power, lumen diameter, and distal flexibility could further optimize the device design according to stone burden and anatomical variability. Additionally, as the thulium fiber laser (TFL) technology has become increasingly prevalent in endourology [37], investigating the potential synergistic benefits of combining TFL with FANS-UAS may lead to further improvements in procedural efficiency. Beyond these aspects, in endourologic stone surgery, procedural complexity is determined not only by stone size but also by the anatomical characteristics of the renal pelvis and calyceal system, as well as the precise stone location. Considerable efforts have been made to develop systematic scoring systems for classifying these anatomical and technical challenges. Importantly, if FANS-UAS demonstrates superior SFR and reduced complication profiles compared with conventional UAS, even in procedures categorized as highly complex, it could constitute a pivotal factor guiding surgeons in selecting the most appropriate operative approach. Finally, considering that suction intensity and sheath flexibility may affect ureteral mucosal integrity, long-term imaging and functional follow-up studies are required to ensure that delayed complications, such as ureteral strictures or vesicoureteral reflux, do not occur.

## 5. Conclusions

This updated meta-analysis demonstrates that FANS-UAS significantly enhances SFR and reduces complications compared with conventional UAS. These benefits are consistent across Asian and Western cohorts, randomized and observational designs, and various laser systems. Importantly, FANS-UAS achieves these improvements without extending operative time or hospital stay, confirming both its efficacy and procedural safety. However, given the methodological limitations of the available evidence, including device heterogeneity, the predominance of retrospective studies, and relatively short follow-up periods, these results should be interpreted with caution. Further well-designed multicenter randomized trials with long-term follow-up are required to confirm the clinical role of FANS-UAS in endourological practice.

## Figures and Tables

**Table 1:** Characteristics of included studies.

Author Year	Country	Design	Procedure	No. Patients	Mean Age (Years)	Stone Size (mm)	Stone Location	SFR Definition	Quality Assessment
Cacciatore et al. 2025 [10]	Italy	RCT	FANS-UAS	62	63.3 (48.7–71.5)	10.5 (8.0–13.0)	Renal	<3 mm fragments	1−
			Conventional UAS	70	57.1 (47.5–68.3)	10.0 (8.0–12.25)			
Zhu et al. 2024 [18]	Multicenter (China, Turkey, Malaysia, Philippines)	RCT	FANS-UAS	160	53.0 (45.0–64.0)	14.0 (10.0–20.0)	Renal, Proximal Ureter	<2 mm fragments	1+
			Conventional UAS	160	52.0 (40.0–61.8)	11.0 (9.0–16.0)			
Yu et al. 2024 [16]	China	Retrospective	FANS-UAS	152	51.1 ± 12.2	15.5 ± 2.0	Renal, Proximal Ureter	Zero fragments	2+
			Conventional UAS	152	50.5 ± 11.8	15.2 ± 1.9			
Ong et al. 2024 [14]	Multicenter (Singapore, UK, Canada, Saudi Arabia, India, Indonesia, Russia, Hong Kong, Italy, France)	Retrospective	FANS-UAS	45	52.0 (40–67)	14 (13–19)	Renal	<4 mm fragments	2+
			Conventional UAS	45	52.0 (39–61)	13 (11–16)			
Chen et al. 2024 [11]	China	Retrospective	FANS-UAS	125	45.6 ± 12.9	28.1 ± 15.6	Renal	<2 mm fragments	2−
			Conventional UAS	113	46.4 ± 14.9	26.8 ± 14.2			
Uslu et al. 2024 [15]	Turkey	Prospective	FANS-UAS	43	56.0 (39.0–69.0)	10 (8–17)	Renal	<3 mm fragments	2+
			Conventional UAS	45	55.0 (42.0–61.0)	11 (8–13)			
Zhang et al. 2023 [17]	China	Retrospective	FANS-UAS	102	46.8 ± 11.9	18.47 ± 4.67	Renal	<2 mm fragments	2−
			Conventional UAS	112	47.7 ± 9.2	18.20 ± 4.46			
Ding et al. 2023 [12]	China	Retrospective	FANS-UAS	138	55.7 ± 13.1	13.0 ± 6.9	Renal	<2 mm fragments	2−
			Conventional UAS	61	57.6 ± 13.7	13.4 ± 5.2			
Huang et al. 2023 [13]	China	Retrospective	FANS-UAS	103	54.7 ± 10.7	17 ± 6	Renal, Proximal Ureter	<3 mm fragments	2+
			Conventional UAS	103	54.5 ± 11.0	17 ± 5			

FANS, flexible and navigable suction ureteral access sheath; UAS, ureteral access sheath; SFR, stone-free rate; RCT, randomized controlled trial. A quality assessment was performed using the Scottish Intercollegiate Guidelines Network (SIGN) checklist. A score of 1+ indicates well-conducted RCT with a low risk of bias; 1 indicates an RCT with a high risk of bias; 2+ indicates well-conducted cohort studies with a low risk of bias; and 2− means cohort studies with a high risk of bias.

**Table 2:** The MINORS scores of the non-randomized studies included in the review.

	Yu et al. 2024 [16]	Ong et al. 2024 [14]	Chen et al. 2024 [11]	Uslu et al. 2024 [15]	Zhang et al. 2023 [17]	Ding et al. 2023 [12]	Huang et al. 2023 [13]
**A clearly** **stated aim**	2	2	2	2	2	2	2
**Inclusion of consecutive samples**	1	2	1	2	1	1	2
**Prospective collection of data**	0	0	0	1	0	0	0
**Endpoints appropriate to the aim of the study**	2	2	2	2	2	2	2
**Unbiased assessment of the study endpoint**	1	1	1	1	1	1	1
**Follow-up period appropriate to the aim of the study**	2	2	2	2	2	2	2
**Loss to follow up less than 5%**	2	2	2	2	2	2	2
**Prospective calculation of the study size**	0	0	0	2	0	0	0
**An adequate control group**	2	2	2	2	2	2	2
**Contemporary groups**	2	2	2	2	2	2	2
**Baseline equivalence of groups**	2	2	2	2	2	2	2
**Adequate statistical analyses**	2	2	1	2	1	2	2
**Total**	18	19	17	22	17	18	19

MINORS, Methodological Index for Non-Randomized Studies. Each item received a score of 0 (not reported), 1 (reported but inadequate), or 2 (reported and adequate). The ideal (maximum) total MINORS score is 16 for noncomparative studies and 24 for comparative studies. MINORS, methodological index for non-randomized studies.

## Data Availability

The data presented in this study are available in the article.

## Supplementary Materials

The following supporting information can be downloaded at: https://www.mdpi.com/article/10.3390/medicina62030536/s1, Table S1: PRISMA checklist [38].
